# Formation of Worm-Like Micelles in Mixed *N*-Hexadecyl-*N*-Methylpyrrolidinium Bromide-Based Cationic Surfactant and Anionic Surfactant Systems

**DOI:** 10.1371/journal.pone.0102539

**Published:** 2014-07-14

**Authors:** Caili Dai, Zhihu Yan, Qing You, Mingyong Du, Mingwei Zhao

**Affiliations:** 1 School of Petroleum Engineering, China University of Petroleum (Huadong), Qingdao, Shandong, P. R. China; 2 School of Energy Resources, China University of Geoscience, Beijing, P. R. China; University of Michigan, United States of America

## Abstract

Through the descriptive and rheological characterization of worm-like micelles formed by *N*-hexadecyl-*N*-methylpyrrolidinium bromide and sodium laurate, the formation and properties of the worm-like micelles were affected by the concentrations of sodium laurate and temperature. Additionally, cryogenic transmission electron microscopy images further validated the formation of worm-like micelles.

## Introduction

Numerous self-aggregates, such as micelles, bicelles, micro emulsions, liquid crystals and vesicles, are able to form by the association of surfactant molecules [1]–[5]. Among these diverse microstructures, worm-like micelles have attracted extensive attention because of excellent performance in terms of viscoelastic behavior [6], [7]. Worm-like micelles have found applications in various areas over the past two decades, such as clear fracturing fluids, heat-conducting fluids, personal care products and templates for nanomaterial synthesis [8]–[11].

In dilute solutions, surfactants generally form small spherical micelles spontaneously when the concentration of surfactant is higher than the critical micelle concentration (CMC) [12], [13]. Concurrently, under appropriate conditions of surfactant concentration, salinity and temperature, the spherical micelles may grow in one-dimension and form entangled worm-like structures that present viscoelastic performance similar to that of polymer solutions [14], [15]. In agreement with the Israelachvili prediction [16], the introduction of additives facilitates the self-assembly of surfactants *via* electrostatic repulsion force screening [17], [18]. However, in contrast to polymers that are connected by covalent bonds, worm-like micelles are also known as “living polymer systems” [19] because they join together through ionic bonds and can break and intertwine reversibly.

Ionic liquids (ILs) are also known as organic molten electrolytes due to their distinctive physical and chemical properties, such as high conductivity, low melting temperature, outstanding catalytic properties, and so on. ILs, therefore, have earned close attention from scientific researchers. Similar to ionic surfactants, surface active ionic liquids (SAILs) exist as a hydrophilic head and a hydrophobic chain simultaneously; hence, the self-assembled aggregates can be formed in aqueous solution. In recent years, the aggregation behaviors of SAILs and their structures, which can be manipulated by changing the cations, anions, substituent, *etc.*, have been reported frequently [20], [21]. Zhao and Zheng synthesized *N-*alkyl-*N-*methylpyrrolidinium bromide (C_n_MPBr) and studied its multiple assembly behaviors [22], [23]. Dong and Zheng synthesized *N-*alkyl-*N-*methylimidazolium bromide (C_n_mimBr) and investigated its phase behavior [24]. Geng and Zheng studied the mutual interaction effects between bovine serum albumin and *N-*tetradecyl-*N-*methylimidazolium bromide [25].

**Figure 1:**
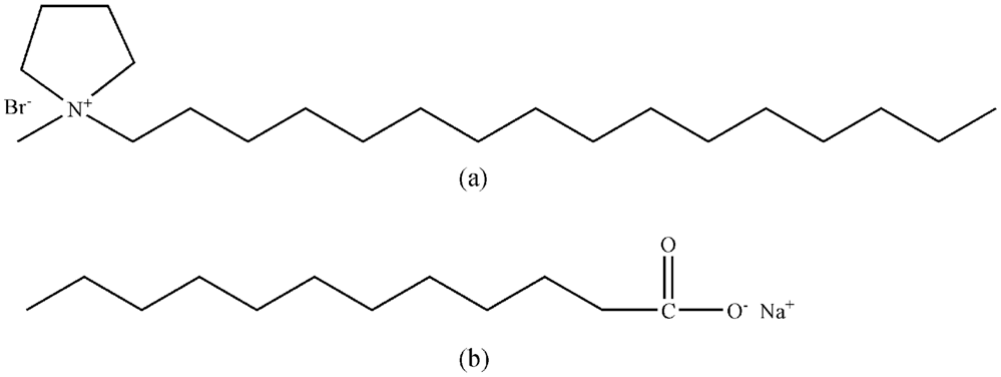
Chemical structure of C_16_MPBr (a) and Sodium Laurate (b).

Therefore, we were motivated to explore worm-like micelles formed by C_16_MPBr in the presence of sodium laurate (SL). Their molecular structures of C_16_MPBr and SL are shown in Figure 1. To study the shape and viscoelastic properties of worm-like micelles, rheometer and cryogenic-transmission electron microscopy (cyro-TEM) were utilized. These techniques were used to observe microscopic configuration, to calculate the rheological parameters and the flow activation energy. In addition, the effect of temperature on the morphology of worm-like micelles were also studied.

## Materials and Methods

### Materials

Cationic surfactant C_16_MPBr was synthesized and purified as described previously [22], [23]. Anionic surfactant SL was an AR grade product of the Aladdin Chemistry Company and was used without purification. Deionized water was used to prepare all solutions.

### Sample preparation

The solutions to be analyzed were prepared by simply mixing a variable concentration of SL with a fixed concentration of C_16_MPBr (70 mM). Samples were homogenized by mild heating and vortex mixed and then placed in a thermostatic bath at 25°C for at least one week to equilibrate before investigation.

### Rheological measurements

The rheological properties were performed on a Physica MCR301 rheometer made by Anton Paar GmbH with a Rotor CC27 system. The shearing rate ranged from 0.01 to 1000 s^−1^ in the steady shear experiment. For the dynamic oscillatory measurements, the frequency region was set to a range of 0.01–100 rad·s^−1^, and the linear viscoelastic region was identified depending on a dynamic strain sweep test, in which the frequency was fixed at 1.0 Hz.

The Maxwell-fluid model with a single relaxation time was usually used to explain the aggregation behavior of worm-like micelle solutions. The elastic modulus (storage modulus) *G′*, the viscous modulus (loss modulus) *G″* and the complex viscosity 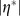 are given by the following equations [1], [2]:
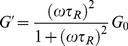
(1)

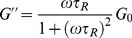
(2)

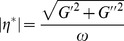
(3)Here, *ω* is the frequency. 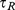 is the relaxation time and is approximately equal to the reciprocal of 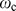, which is the crossover frequency when *G′* and *G″* intersect. *G_0_* is the plateau modulus, which is estimated by reaching a plateau at high frequency.

### Cryo-TEM

Specimens for examination were stored in a controlled environment vitrification system with temperature and humidity control functions. The preparation of the cryo-TEM samples was performed with the following sequence of operations. First, 5 mL of the sample was placed onto a perforated polymer film held by tweezers to insure that the formation of the thin film spanned the mesh hole. Second, after 10 seconds, the samples were immediately immersed into liquid ethane just above its freezing point of −183°C. Third, the samples were then stored in liquid nitrogen to protect against contamination before they were moved to a Gatan cryo Holder 626 and examined with a FEI Tecnai 20 TEM (200 kV) at about −174°C. The images were captured with a Gatan US1000 894 CCD and processed with Leginon software.

## Results and Discussion

### The formation of worm-like micelles

**Figure 2:**
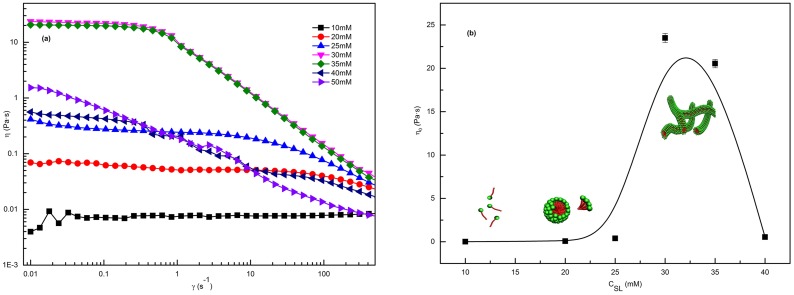
Steady rheology plots. (a) The shear viscosity for 70 mM C_16_MPBr with different SL concentrations at *T* = 25°C; (b) Variations of zero-shear viscosity (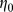) as a function of different SL concentrations for the 70 mM C_16_MPBr. The error bars represent standard deviations.

The shear viscosity of 70 mM C_16_MPBr with different concentrations of SL is measured by the rheometer and the results are plotted in Figure 2a. At low SL concentrations, the samples show the typical characteristics of a Newtonian fluid; the steady-shear viscosity does not change with the shear rate. When the concentration of SL exceeds 20 mM, the samples show a non-Newtonian flow phenomenon; the viscosity maintains a constant value (Newtonian plateau value) at low shear rates and decreases dramatically at high shear rates, deviating from the simple Maxwell Model. Flow instability resulting in shear-banding that occurred in the solutions is the main reason why differences appeared from low to high shear rates. Usually, this phenomenon is identified as evidence of the formation of worm-like micelles [26]. When the concentration of SL exceeds 50 mM, the color of the solutions change from transparent to milky due to the formation of precipitate, which is generally called the salting-out effect [12].

The variation of zero-shear viscosity 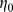, which can be calculated from the average viscosity of the low shear rate, as a function of SL concentration is shown in Figure 2b. At the beginning, the values of 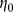 are approximately zero, indicating Newtonian fluid behavior. With the increase of SL concentration, the 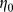 decreases sharply after reaching the peak value of 23.5 Pa·s at 30 mM. The value of the maximum 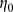 was typical for SAILs. An increase in the curvature energy of surfactant aggregates is induced by an increase in the salt concentration, causing the flexible worm-like micelles to form from the original spherical micelles, which schematic illustrate in figure 2b. The decrease in viscosity is generally attributed to a faster time-scale for reversible breakage which allows disentanglement to happen faster and the formation of branched micelles [27].

### Rheological model of worm-like micelles

**Figure 3:**
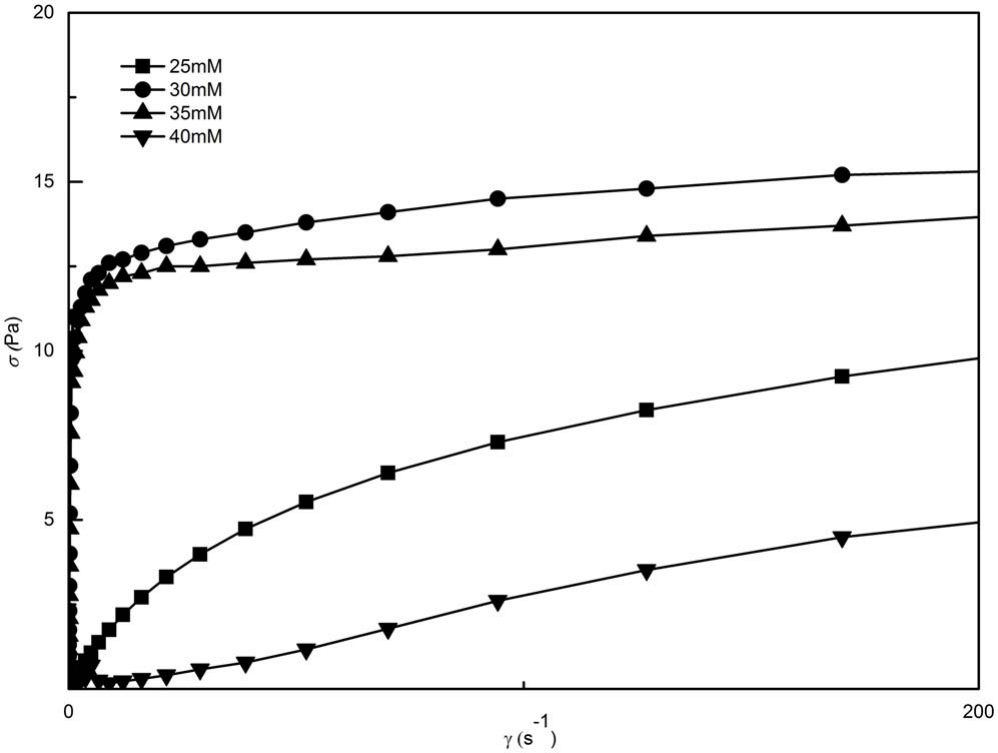
Shear stress with different concentration of SL at a fixed C_16_MPBr concentration of 70 Mm.

The variation profile of shear stress *σ* on the shear rate *γ* with different concentrations of SL at 70 mM C_16_MPBr is shown in Figure 3. The worm-like micellar solutions belong to the class of viscoelastic fluids with no yield stress, which can be inferred from the fact that all curves pass through the origin and show a linear relationship [28].

### The viscoelasticity of worm-like micelles

To characterize the viscoelastic behavior, dynamic measurements were employed to test the C_16_MPBr/SL solution system. Before dynamic rheological experiments, the linear viscoelastic zone where G* (complex modulus) is independent of the applied stress was first determined through the stress sweep measurement. *σ* = 1.0 Pa was chosen in subsequent frequency sweep measurements according to the stress scanning of the 70 mM C_16_MPBr/30 mM SL solution.

**Figure 4:**
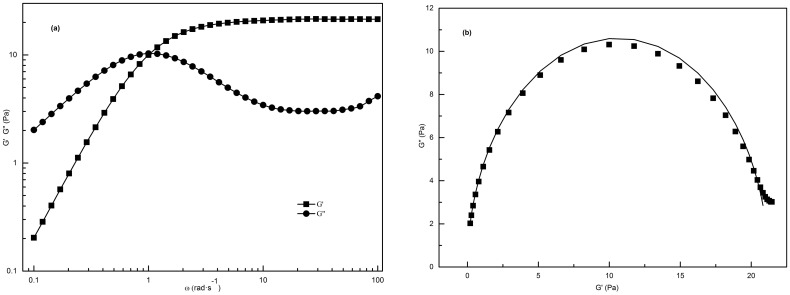
Dynamic oscillatory plots. (a) Variations of G′ and G″ as a function of frequency (*ω*) in aqueous 70 mM C_16_MPBr/30 mM SL solution; (b) Cole-Cole plots (solid lines indicate the best fitting of Maxwell model).

Figure 4a shows the frequency (*ω*) dependence of the storage (*G′*) and loss (*G″*) modulus for the sample containing 70 mM C_16_MPBr/30 mM SL. With the increase of *ω,* both *G′* and *G″* increased in the low frequency region, and *G′* continued to rise, while *G″* declined after the crossover frequency 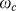. The sample shows liquid-like behavior (*G′*<*G″*) before 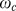 and solid-like behavior (*G′*>*G″*) thereafter. Finally, *G′* reaches a plateau (*G_0_*), while *G″* increases again after reaching a minimum (*G″_min_*). This trend fits with the viscoelastic characteristics of worm-like micelles that follow the single-element Maxwell’s model [29], [30]. The moduli *G′* and *G″* vary as *ω^2^* and *ω* in the low frequency region, and deviation from the rules occurs in the high frequency region. These are two pieces of evidence that the formation of worm-like micelles and the deviation can be explained by breaking/recombining or the dynamic equilibrium theory [31].

To uncover how well the data are in accord with the semicircle characteristic of the Maxwell-fluid model, “Cole-Cole” plots of *G″* versus *G′* are described by [6]:
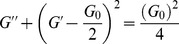
(4)


As shown in Figure 4b, the shape of the curve follows semicircular behavior perfectly, which indicates the formation of worm-like micelles at low and medium frequency, whereas the degree of deviation from the semicircular behavior at high frequency can be obtained by measuring the deviation of the data points.

**Figure 5:**
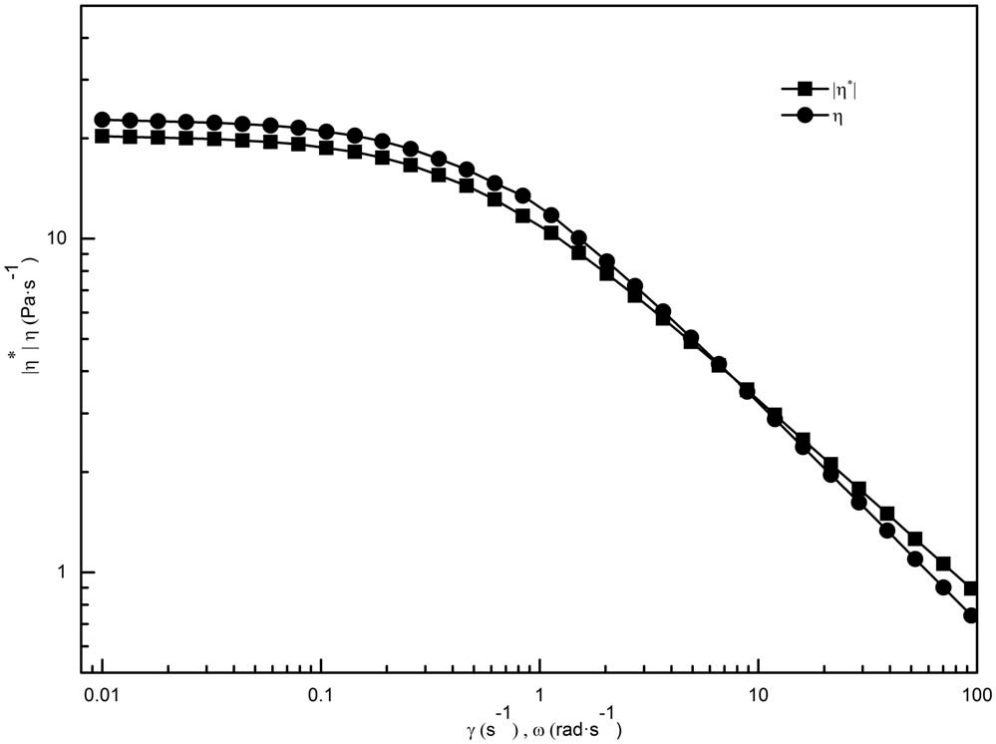
Shear-rate dependence of the steady-shear viscosity and frequency dependence of the complex viscosity for 70 mM C_16_MPBr/30 mM SL solution.

In general, the steady-shear viscosity (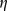) and the complex viscosity (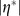) of surfactant solutions with network structures, such as worm-like micelles, are equal or near-equal when shear rate (*γ*) and frequency (*ω*) are equivalent values. When 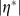 is significantly larger than 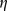, if the structures can survive small oscillatory deformations but are ruptured by large deformations, we can draw the conclusion that the Cox–Merz empirical rule does not apply. The steady-shear viscosity as a function of 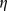 and the complex viscosity as a function of 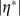 for the 70 mM C_16_MPBr/30 mM SL solution is shown in Figure 5. The coincidence of 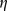 and 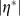 indicates that worm-like micelles that possess rigid network structure have already formed [32]. The curves deviate from the Cox–Merz empirical rule at high frequencies, a result that can be attributed to faster breakdown and reconnection of the aggregates.

The rheological parameters related to the microstructures of C_16_MPBr solutions with different SL concentrations have been calculated according to the following formulas [1], [2], and the results are listed in Table 1.
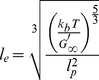
(5)

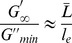
(6)

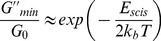
(7)


**Table 1:** Various rheological parameters calculated for the aqueous solutions with different SL concentration at a fixed concentration of 70_16_MPBr.

SL(mM)	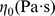	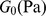	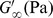	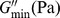	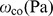	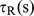	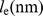	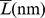
30	22.95±0.32	21.22±0.25	20.61±0.21	3.01±0.007	1.03±0.02	0.97±0.02	145±1.2	991±31.67
35	20.55±0.23	21.03±0.18	20.38±0.22	2.28±0.008	0.95±0.03	1.056±0.03	146±1.2	1304±24.57

The errors reported here were calculated from the standard deviation for all the measurements.

Here, *l_e_* is the entanglement length, *l_p_* is the persistence length, 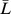 is the average contour length, *k_b_* is the Boltzmann constant, *T* is the absolute temperature, 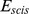 is the scission energy, and 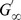 is obtained by the extrapolation of the Cole-Cole plots at high frequencies to the X-axis intercept or from the equation 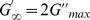, where 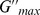 is the viscosity modulus at shear frequency *ω_c_.* Here, we set *l_p_* = 15 nm according to the literature [28]. From Table 1, it can be observed that 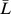 is largest and *ω_c_* is smallest for the sample at 35 mM SL. The 70 mM C_16_MPBr/35 mM SL solution has the longest worm-like micelles and the longest kinetic lifetimes [2].

### Effect of temperature

We also studied whether temperature has an impact on the formation of worm-like micelles. To show this effect quantitatively, rheological measurements of the 70 mM C_16_MPBr/30 mM SL solution at different temperatures, from 15°C to 30°C, and related rheological parameters are calculated in Table 2. The values of 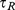, 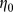 and 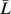 clearly decrease, but *G_0_* alone and *l_e_* are constant with temperature, albeit in an opposite manner. In the case of heating, the constant of *G_0_* indicates that the degree of entanglement of the micellar network is unchanged, and a decline of 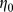 shows a lower viscosity of the sample. We speculate that the viscosity decrease is not only on account of the decrease of 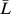 but also because of the amount of branching joints growing as a consequence of the rise in temperature. The growth of branching joints aggravate slide effects that accompany applied force, hence allowing a fast relaxation process and a decrement in viscosity under the condition of a maintained network structure [33].

**Table 2:** Various rheological parameters calculated for the sample of 70_16_MPBr/30 mM SL at different temperatures.

*T*(°C)	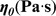	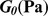	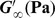	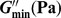	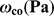	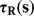	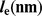	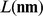
15	80.63±2.17	20.71±0.62	17.97±0.43	1.97±0.09	0.18±0.003	5.46±0.004	153±2.94	1394±70.77
20	50.31±1.03	20.74±0.57	19.52±0.54	2.38±0.12	0.44±0.02	2.25±0.07	148±1.23	1210±80.68
25	22.95±0.54	21.22±0.61	20.61±0.61	3.01±0.14	1.03±0.04	0.97±0.04	145±3.22	991±47.98
30	11.67±0.23	20.33±0.59	20.54±0.57	3.13±0.13	2.21±0.08	0.45±0.01	146±3.33	959±38.42

The errors reported here were calculated from the standard deviation for all the measurements.

**Figure 6:**
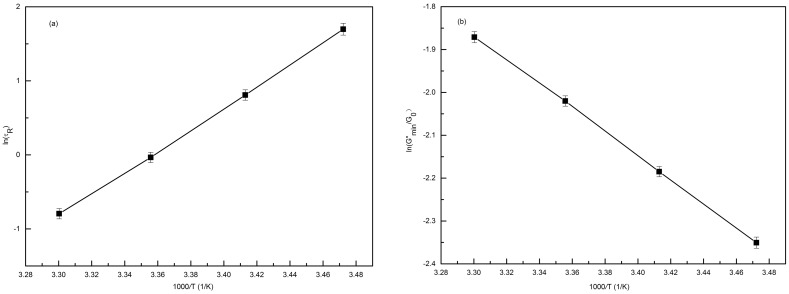
Temperature effect plots. (a) An Arrhenius plot of ln*τ*_R_ as a function of 1/T for the 70 mM C_16_MPBr/30 mM SL solution; (b) the plot of ln (G″_min_/G_0_) as a function of 1/T. The error bars represent standard deviations.

The relaxation time fits the Arrhenius equation [34], resulting in a linear relationship, which can be observed from Figure 6a, which shows an Arrhenius plot of ln 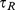 versus 10^3^/*T* as given by [1], [2]:
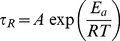
(8)Here, *E_a_* is the flow activation energy, *R* is the gas constant and *A* is a constant. The value of *E_a_* calculated from the slope is approximately 120****kJ·mol^−1^, approximately equal to the values of worm-like micelle systems reported previously [35]. Figure 6b presents the plot of ln (*G″_min_*/*G_0_*) as a function of the reciprocal of the absolute temperature, according to Eq. (7). The value of 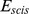 is equal to 228****kJ·mol^−1^ for the 70 mM C_16_MPBr/30 mM SL solution indicates that it is more favorable to form elongated micelles with kinetic lifetime longer than conventional ionic surfactants such as CTAB [2], [12].

### Cryo-TEM

**Figure 7:**
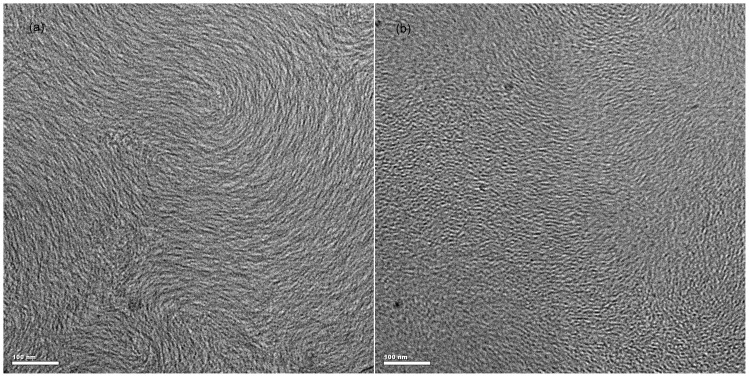
Cryo-TEM images. (a) 70 mM C_16_MPBr/30 mM SL solution; (b) 70 mM C_16_MPBr/35 mM SL solution.

Cryo-TEM is employed for the direct observation of the micelle morphology and micelle joints of C_16_MPBr/SL solutions. As above, the morphology of aggregation undergoes a transformation process from spherical aggregates to worm-like micelles. During this process, the value of 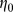 achieves a maximum value. Figure 7a and Figure 7b show that three-dimensional networks of worm-like micelles are observed in the 70 mM C_16_MPBr/30 mM SL solution and 70 mM C_16_MPBr/35 mM SL solution, respectively. The contour length could not be obtained on account of the unclear ends of the micelles. These worm-like micelles are found to overlap with each other and entangle into three-dimensional network structures, which corroborate the results of the rheology measurements, *i.e.*, the appearance of a maximum 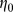 is attributed to the presence of worm-like micelles. Other than the entangled worm-like micelles, a few branched micelles with joints were also observed that can explicitly explain the temperature effect on the character of worm-like micelles.

## Conclusions

In summary, with the aid of sodium laurate, the aggregation behavior of worm-like micelles that fit the Maxwell model of a single stress relaxation mode has been observed in aqueous solution. These were formed by a novel surface active IL, *N*-hexadecyl-*N*-methylpyrrolidinium bromide. Rheological measurements revealed that the main factor affecting the viscoelastic properties of worm-like micelles are temperature and the concentrations of sodium laurate. Cryo-TEM imaging visually observed the formation of worm-like micelles and verified the influencing factors on the network structure of worm-like micelles. We believe that this work further explained the mechanism of formation for solutions of worm-like micelles with high viscoelasticity and serves as a possible utilization of ILs in colloidal systems.

## References

[1] Cates ME, Candau S (1990) Statics and dynamics of worm-like surfactant micelles. Journal of Physics: Condensed Matter 2: 6869.

[2] Rehage H, Hoffmann H (1991) Viscoelastic surfactant solutions: model systems for rheological research. Molecular Physics 74: 933–973.

[3] Dvinskikh SV, Dürr UH, Yamamoto K, Ramamoorthy A (2007) High-resolution 2D NMR spectroscopy of bicelles to measure the membrane interaction of ligands. Journal of the American Chemical Society 129: 794–802. 17243815 10.1021/ja065536k PMC2527737

[4] Chu Z, Dreiss CA, Feng Y (2013) Smart wormlike micelles. Chemical Society Reviews 42: 7174–7203. 23545844 10.1039/c3cs35490c

[5] Anderson V, Pearson J, Boek E (2006) The rheology of worm-like micellar fluids. Rheology reviews 2006: 217–253.

[6] Cates ME, Fielding SM (2006) Rheology of giant micelles. Advances in Physics 55: 799–879.

[7] Granek R, Cates ME (1992) Stress relaxation in living polymers: Results from a Poisson renewal model. The Journal of Chemical Physics 96: 4758.

[8] Xu J, Dürr UH, Im SC, Gan Z, Waskell L, et al (2008) Bicelle-Enabled Structural Studies on a Membrane-Associated Cytochrome b5 by Solid-State MAS NMR Spectroscopy. Angewandte Chemie 120: 7982–7985. 10.1002/anie.200801338 PMC2936958 18792050

[9] Dvinskikh SV, Yamamoto K, Dürr UH, Ramamoorthy A (2007) Sensitivity and resolution enhancement in solid-state NMR spectroscopy of bicelles. Journal of Magnetic Resonance 184: 228–235. 17084096 10.1016/j.jmr.2006.10.004 PMC1861833

[10] Yamamoto K, Soong R, Ramamoorthy A (2009) Comprehensive analysis of lipid dynamics variation with lipid composition and hydration of bicelles using nuclear magnetic resonance (NMR) spectroscopy. Langmuir 25: 7010–7018. 19397253 10.1021/la900200s PMC2794801

[11] Dvinskikh S, Dürr U, Yamamoto K, Ramamoorthy A (2006) A high-resolution solid-state NMR approach for the structural studies of bicelles. Journal of the American Chemical Society 128: 6326–6327. 16683791 10.1021/ja061153a PMC2529225

[12] Davies TS, Ketner AM, Raghavan SR (2006) Self-assembly of surfactant vesicles that transform into viscoelastic wormlike micelles upon heating. Journal of the American Chemical Society 128: 6669–6675. 16704268 10.1021/ja060021e

[13] Hoffmann H, Rauscher A, Gradzielski M, Schulz S (1992) Influence of ionic surfactants on the viscoelastic properties of zwitterionic surfactant solutions. Langmuir 8: 2140–2146.

[14] Rehage H, Hoffmann H (1988) Rheological properties of viscoelastic surfactant systems. The Journal of Physical Chemistry 92: 4712–4719.

[15] Moitzi C, Freiberger N, Glatter O (2005) Viscoelastic wormlike micellar solutions made from nonionic surfactants: structural investigations by SANS and DLS. The Journal of Physical Chemistry B 109: 16161–16168. 16853053 10.1021/jp0441691

[16] Israelachvili JN (2011) Intermolecular and surface forces: revised third edition: Academic press.

[17] Shikata T, Hirata H, Kotaka T (1987) Micelle formation of detergent molecules in aqueous media: viscoelastic properties of aqueous cetyltrimethylammonium bromide solutions. Langmuir 3: 1081–1086.

[18] Candau S, Hirsch E, Zana R, Adam M (1988) Network properties of semidilute aqueous KBr solutions of cetyltrimethylammonium bromide. Journal of colloid and interface science 122: 430–440.

[19] Candau S, Hirsch E, Zana R (1985) Light scattering investigations of the behavior of semidilute aqueous micellar solutions of cetyltrimethylammonium bromide: analogy with semidilute polymer solutions. Journal of colloid and interface science 105: 521–528.

[20] Dong B, Li N, Zheng L, Yu L, Inoue T (2007) Surface adsorption and micelle formation of surface active ionic liquids in aqueous solution. Langmuir 23: 4178–4182. 17346069 10.1021/la0633029

[21] Welton T (1999) Room-temperature ionic liquids. Solvents for synthesis and catalysis. Chemical reviews 99: 2071–2084. 11849019 10.1021/cr980032t

[22] Zhao M, Zheng L (2011) Micelle formation by N-alkyl-N-methylpyrrolidinium bromide in aqueous solution. Physical Chemistry Chemical Physics 13: 1332–1337. 21103577 10.1039/c0cp00342e

[23] Shi L, Zhao M, Zheng L (2012) Lyotropic liquid crystalline phases formed in ternary mixtures of N-alkyl-N-methylpyrrolidinium bromide/1-decanol/water. RSC Advances 2: 11922–11929.

[24] Dong B, Zhao X, Zheng L, Zhang J, Li N, et al (2008) Aggregation behavior of long-chain imidazolium ionic liquids in aqueous solution: micellization and characterization of micelle microenvironment. Colloids and Surfaces A: Physicochemical and Engineering Aspects 317: 666–672.

[25] Geng F, Zheng L, Yu L, Li G, Tung C (2010) Interaction of bovine serum albumin and long-chain imidazolium ionic liquid measured by fluorescence spectra and surface tension. Process biochemistry 45: 306–311.

[26] Ali AA, Makhloufi R (1999) Effect of organic salts on micellar growth and structure studied by rheology. Colloid and Polymer Science 277: 270–275.

[27] Lin Z (1996) Branched worm-like micelles and their networks. Langmuir 12: 1729–1737.

[28] Li J, Zhao M, Zheng L (2012) Salt-induced wormlike micelles formed by *N*-alkyl-*N*-methylpyrrolidinium bromide in aqueous solution. Colloids and Surfaces A: Physicochemical and Engineering Aspects 396: 16–21.

[29] Lu T, Huang J, Li Z, Jia S, Fu H (2008) Effect of hydrotropic salt on the assembly transitions and rheological responses of cationic gemini surfactant solutions. The Journal of Physical Chemistry B 112: 2909–2914. 18275179 10.1021/jp0766205

[30] Acharya DP, Hossain MK, Sakai T, Kunieda H (2004) Phase and rheological behaviour of viscoelastic wormlike micellar solutions formed in mixed nonionic surfactant systems. Physical Chemistry Chemical Physics 6: 1627–1631.

[31] Pei X, Zhao J, Wei X (2011) Wormlike micelles formed by mixed cationic and anionic gemini surfactants in aqueous solution. Journal of colloid and interface science 356: 176–181. 21276973 10.1016/j.jcis.2010.12.065

[32] Magid L (1998) The surfactant-polyelectrolyte analogy. The Journal of Physical Chemistry B 102: 4064–4074.

[33] Shrestha RG, Shrestha LK, Aramaki K (2007) Formation of wormlike micelle in a mixed amino-acid based anionic surfactant and cationic surfactant systems. Journal of colloid and interface science 311: 276–284. 17368470 10.1016/j.jcis.2007.02.050

[34] Kumar R, Kalur GC, Ziserman L, Danino D, Raghavan SR (2007) Wormlike micelles of a C22-tailed zwitterionic betaine surfactant: from viscoelastic solutions to elastic gels. Langmuir 23: 12849–12856. 18004899 10.1021/la7028559

[35] Shrestha RG, Tobita K, Aramaki K (2009) Rheological behavior of viscoelastic wormlike micelles in mixed *N*-dodecyl glutamic acid/poly (oxyethylene) hexadecyl ether systems in presence of salts. Colloids and Surfaces A: Physicochemical and Engineering Aspects 332: 103–111.

